# Atmospheric Pressure Plasma Polymerisation of D-Limonene and Its Antimicrobial Activity

**DOI:** 10.3390/polym15020307

**Published:** 2023-01-06

**Authors:** Asad Masood, Naeem Ahmed, M. F. Mohd Razip Wee, Anuttam Patra, Ebrahim Mahmoudi, Kim S. Siow

**Affiliations:** 1 Institute of Microengineering and Nanoelectronics, University Kebangsaan Malaysia, Bangi 43600, Selangor, Malaysia; 2 Chemistry of Interfaces Group, Luleå University of Technology, SE-97187 Luleå, Sweden; 3 Department of Chemical and Process Engineering, Faculty of Engineering and Built Environment, Universiti Kebangsaan Malaysia, Bangi 43600, Selangor, Malaysia

**Keywords:** atmospheric pressure, plasma polymerisation, D-limonene, ASTM E2149, antimicrobial coating, *E. coli* bacteria

## Abstract

Antibacterial coating is necessary to prevent biofilm-forming bacteria from colonising medical tools causing infection and sepsis in patients. The recent coating strategies such as immobilisation of antimicrobial materials and low-pressure plasma polymerisation may require multiple processing steps involving a high-vacuum system and time-consuming process. Some of those have limited efficacy and durability. Here, we report a rapid and one-step atmospheric pressure plasma polymerisation (APPP) of D-limonene to produce nano-thin films with hydrophobic-like properties for antibacterial applications. The influence of plasma polymerisation time on the thickness, surface characteristic, and chemical composition of the plasma-polymerised films was systematically investigated. Results showed that the nano-thin films deposited at 1 min on glass substrate are optically transparent and homogenous, with a thickness of 44.3 ± 4.8 nm, a smooth surface with an average roughness of 0.23 ± 0.02 nm. For its antimicrobial activity, the biofilm assay evaluation revealed a significant 94% decrease in the number of Escherichia coli (*E. coli*) compared to the control sample. More importantly, the resultant nano-thin films exhibited a potent bactericidal effect that can distort and rupture the membrane of the treated bacteria. These findings provide important insights into the development of bacteria-resistant and biocompatible coatings on the arbitrary substrate in a straightforward and cost-effective route at atmospheric pressure.

## 1. Introduction

Infectious illness treatment is becoming more complex for physicians due to an increasing rate of antibiotic resistance around the world, causing major morbidity and death. Pharmaceutical companies are insufficiently responding to the rising demand for new antibiotics. Alternative medicinal approaches based on age-old herbal expertise utilising secondary plant compounds as promising antibacterial agents may aid in the development of new drugs [1]. Alkaloids, phenolics, terpenes, and saponins are examples of secondary metabolites known to assist and keep homeostasis in their surroundings [2]. Unlike the present synthetic antibiotics, these phytochemicals are recognised to have a weak antagonistic response while still possessing influential antibacterial properties.

Terpenes are a type of natural substance made up of cycloaliphatic and/or aromatic components. Limonene is one of the members of this family. It is thought to be a promising bioactive chemical since it has antioxidant, anticancer, anti-inflammatory, antiviral, and insecticidal activities. The virucidal activity of SARS-CoV-2 was reduced by about 6 logs in mouthwash that contained both D-limonene and cetylpyridinium chloride (CPC) [3]. Limonene can be used as an antibacterial agent because of its vast variety of medicinal applications. The science of antibacterial properties and mechanisms involved should be constructed to uncover the plausible function of the compounds against diverse germs to investigate natural compounds as medication in a much more practical route. Previous research on the antimicrobial activity of terpenes and their compounds has revealed that sub-lethal disruption to the cytoplasmic membranes is a possible mechanism for *E. coli* death [4,5,6]. Recent findings by Gupta et al. described the different sequential changes that occurred in *E. coli* cells after exposure to limonene, which caused cell rupture and eventually led to cell death [7]. Due to a growing interest in recent implantable medical devices, the issues arising from bacterial infection at the site of the devices have also become a major health challenge [8,9,10,11]. Therefore, there is a great need to produce an effective antimicrobial coating with various surface modifications for contact-killing bacteria on the device surfaces [12,13]. Lately, contact-active antimicrobial coatings have received increasing consideration [14,15].

Thin film technology advancements are critical for a range of research sectors, including microelectronics, biomedical, and anticorrosion applications [16,17,18,19,20]. Nanometre-thick films (<100 nm), for example, can be useful as bioactive layers for implant materials [21] and biomaterial films for anticorrosion purposes [22]. There has been a surge of interest in developing ecologically friendly films from renewable organic precursors [23]. This quest for alternative materials is motivated by a desire to reduce reliance on petroleum-derived resources and to generate goods with higher added value derived from low-cost sources [24]. These are induced via degradative chain transfer processes, which obstruct polymer synthesis [25]. Unlike traditional polymerisation techniques, plasma polymerisation allows thin film deposition from most of the organic precursors [26]. Terpenes originating from plants and fruits, such as terpinen-4-ol [27], geranium [28], and carvone [29] have been shown to create solid films using plasma polymerisation. During the plasma polymerisation reaction, ionic species are bombarded, resulting in radical sites that stimulate cross-linking of the deposited nano-thin films and grafting of new species [30].

Even though limonene has a chemical structure comparable to those terpenes-related precursors, no feasible experiments have been conducted yet using this precursor to make nano-thin films via APPP for antibacterial activity. For instance, a plasma coating with antibacterial surface modifications by an atmospheric pressure plasma jet (APPJ) process is a relatively new field due to its simplicity and convenience of usage [31,32]. In most APPJs and dielectric barrier discharges (DBDs) [33], one electrode is grounded while the other is energised with a high-voltage source to create an electric field between them. According to this method, stable plasma is formed when a flow of gas travels between the coaxial electrodes and is ionised by the electric field. As precursors for the deposited polymer coating, monomers must be vaporised before the plasma ignition. The contact between the precursor and the plasma in the air causes the monomers to fragment and induces the oxygen- and nitrogen-containing groups that dictate the coating characteristics [31]. The products generated from the precursor-plasma reactions, such as reactive plasma species, as well as non-reactive species, deposit on the substrate, where the adsorption and surface reactions take place simultaneously. Some of the published results have been reviewed here [34,35,36,37].

Developing a simple and green route for imparting antibacterial and hydrophobic properties is highly desirable because of economic and environmental reasons. In this work, we reported a rapid and one-step plasma polymerisation technique, utilising D-limonene as a precursor, to create nano-thin films at atmospheric pressure (AP) for antibacterial applications. To the best of our knowledge, the use of APPP of D-limonene as a potential antibacterial agent with a bactericidal effect has not yet been demonstrated. Previous work on the deposition of the D-limonene coating used the low-pressure plasma polymerisation technique approach, which necessitated the use of vacuum [38]. In our APPP approach, the impact of plasma polymerisation time on the physicochemical properties of the resultant nano-thin films was investigated here to demonstrate its feasibility. As an initial proof of concept, the results were incorporated to produce an effective antibacterial coating against Gram-negative (*E. coli*) bacteria, in which *E. coli* exposed to the D-limonene coated surface were evaluated in vitro via field-emission scanning electron microscopy and fluorescence microscopy to access their survivability after incubation.

## 2. Materials and Methods

### 2.1. Materials

The essential oil (D-limonene, C_10_H_16_) was acquired from Sigma Aldrich (St. Louis, MO, USA) with a purity of >97%. It was utilised as received without any additional purification. Microscopic glass slides were selected as a model substrate because of their established record as a positive control. Each glass slide was ultrasonically cleaned for 30 min in acetone, ethanol, deionised (DI) water, and lastly in isopropyl alcohol (IPA). For plasma discharge, 99.99% pure Ar gas was employed in this work.

### 2.2. Experimental Setup of the AP Plasma System

**Figure 1:**
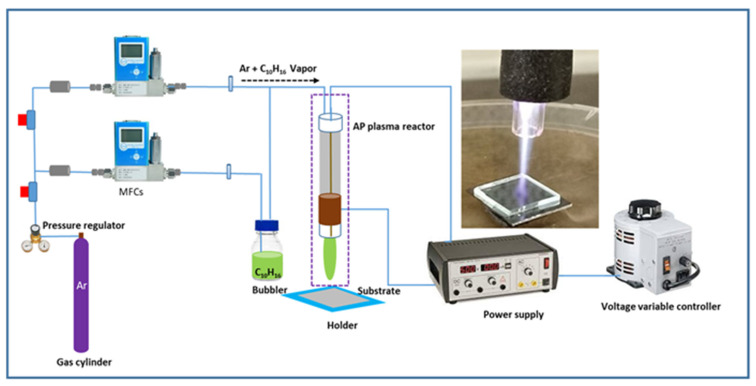
Schematic diagram of the APPP setup. The inset shows the photograph of plasma generated from the APPJ during the deposition process.

Figure 1 shows a schematic diagram of the AP plasma polymerisation system where the plasma discharge was created in a plasma jet consisting of a quartz tube. A copper rod was placed into the quartz tube connected to a high voltage (HV) supply and acted as an HV electrode. The outside electrode, fastened around the quartz tube, serving as a grounded electrode, was likewise made of copper. A neon power supply with a 3 kV output voltage was used to power up the plasma jet. The inner and outer diameters of quartz tubes were about 3.0 and 5.0 mm, respectively. The distance between the HV electrode tip and the nozzle was maintained at 20 mm throughout the experiment, whereas the nozzle was 15 mm away from the target glass substrate. The deposition of AP plasma-polymerised D-limonene (AP-PP-lim) was carried out on the glass substrates of 5 cm × 5 cm size. Mass flow controllers (MFCs) were used to feed the argon into the system. To vaporise the liquid D-limonene monomer, the Ar gas was supplied into a glass bubbler at a constant flow rate of 130 sccm.

### 2.3. Characterisation of AP-PP-lim Nano-Thin Films

The static water contact angle (WCA) measurements were carried out on an automated Contact Angle Goniometer (Rame−Hart, Inc. model 100, Succasunna, NJ, USA) using a static sessile drop technique at room temperature. Two microlitre droplets of DI water were carefully placed on the samples and WCAs were recorded. The surface masking approach was utilised to determine the thickness of the AP-PP-lim nano-thin films on glass substrate [29]. The step height was measured with a surface profiler (model: Bruker Dektak XT). An atomic force microscopy (AFM) system (NT-MDT, Automated AFM NEXT) operating in non-contact mode was used to assess the roughness of the AP-PP-lim. A non-contact silicon gold cantilever beam (NSG-10), with a resonance frequency of 390 kHz and a force constant of 37 Nm^−1^, was used, and the average roughness (*R*_a_) for the scan regions of 10 μm × 10 μm was also measured.

The chemical composition of the pure D-limonene and AP-PP-lim samples was assessed using a Perkin Elmer Spectrum 400 FT-IR Spectrometer (1250 to 4000 cm^−1^ range). Based on 32 accumulated scans with a resolution of 4 cm^−1^, the spectra were acquired. To analyse the spectra, adjust the baseline, and locate the relevant peaks, Spectrum^TM^ 10 software was employed.

Furthermore, the surface chemistry of the samples was analysed using an XPS system (ULVAC-PHI, Quantera II) with an Al-K source that had an energy of 1486 eV and functioned at 50 W and 15 kV. All spectra were acquired with an emission angle of 45°. Each sample was subjected to five scan cycles. Based on the survey scan spectra, the PHI Multipak programme was used to estimate the atomic content of carbon (C) and oxygen (O). For the high-resolution spectra, the peak deconvolutions of C 1 s spectra were fitted using Gaussian curve fitting. During component fitting, the Shirley background was used to deduce the C 1 s spectra, and the full width at half maximum was set between 1.46 and 1.55 eV [39,40].

### 2.4. Microbiological Activity

The antimicrobial activity of AP-PP-lim coatings was evaluated based on the standard test method (ASTM E2149-20). A quantifiable antimicrobial testing technique was used to determine the antibacterial activity of non-leaching antibacterial agents [41]. The antibacterial properties of the coatings were tested using the Gram-negative (*E. coli*) bacteria (ATTC 25927). To grow these bacteria, Luria-Bertani (LB) broth was grown overnight at 37 °C. The inoculum (1 × 10^8^ CFU mL^−1^) and LB media were placed together at a ratio of 1:9 to make an inoculum (1 × 10^7^ CFU mL^−1^) and further diluted to 1 × 10^5^ CFU mL^−1^. *E. coli* bacteria were streaked onto agar (nutrient) plates and left overnight at 37 °C. The samples were placed into 12-well plates and incubated with inoculum (1 × 10^5^ CFU mL^−1^) for 24 h for biofilm formation studies. The Gram staining protocol was used to identify the Gram-negative bacteria by colouring the cells [42]. In a nutshell, the samples were steeped in crystal violet solution for 30 s before being rinsed in saline and then submerged in Gram iodine solution for another 30 s. The weak colours were subsequently decoloured using a decolouriser (GMO-CO2). The average surface area of bacteria was estimated using ImageJ^®^ software (NIH and LOCI, Wisconsin, USA) after the sample images were optically captured at different spots using a fluorescence microscopy system (Olympus, BX53M). Each experiment was carried out three times. The morphology of the bacteria [43] on the control and treated samples was studied using a field-emission electron scanning microscopy (FESEM) system (Zeiss, Supra 55 VP). The sample preparation method was similar to those used for biofilm formation, with the exception that after 24 h of incubation, the samples were rinsed with saline solution to eliminate any loose biofilms. Lastly, the samples were dried at ≃35 °C for 2 h. Prior to FESEM imaging, a 99.99% platinum ultra-thin coating was applied to reduce the charging effect on the non-conducting sample. A live-dead fluorescence experiment was also used to examine the antibacterial activity of the plasma-polymerised film and 12-well plates were used to hold the prepared samples (AP-PP-lim and clean glass substrate). These samples were treated with an inoculum (1 × 10^7^ CFUmL^−1^) for 24 h at 37 °C. The samples were then preserved with 10% *v*/*v* neutral buffered formalin saline after being rinsed with phosphate-buffered saline (PBS). To determine the bacteria’s viability, they were then stained using the LIVE/DEAD^®^ BaclightTM bacterial viability kit (L7007, Invitrogen, ThermoFisher Scientific, Waltham, MA, USA). Before examining the stained samples under a fluorescence microscope, the samples were submerged in 0.9 *w*/*v* saline water to eliminate any extra staining. An Olympus BX51 fluorescence microscope was used to image the viability of bacteria on the AP-PP-lim and glass substrate at a magnification of 60×. The red fluorescent substance, propidium iodide, was seen at an excitation wavelength of 490 nm and an emission wavelength of 635 nm, whereas the green fluorescent substance, SYTO9, was seen at 480 nm and 500 nm. Each sample received five images from experiments performed in triplicate.

## 3. Results and Discussion

### 3.1. Characteristics of AP-PP-lim Nano-Thin Films

The sessile drops technique was used to test the wettability of plasma-polymerised films. The angle at the triple-phase contact line between the water droplet and the film determined the wettability. The adhesive forces between such a liquid drop and a substrate are theoretically a local response driven by interactions between the actual drop and the nearby vapour with the substrate, demanding drop-volume independence [44]. Droplet symmetry may be affected by the distribution of chemical composition and roughness of the surface [45]. The topography is an important factor to consider since roughness can increase contact angles in some circumstances [46]. Contact angle measurements, on the other hand, offer information regarding the nature of a topmost layer of the surface in the 0.5–1.0 nm range [47].

**Figure 2:**
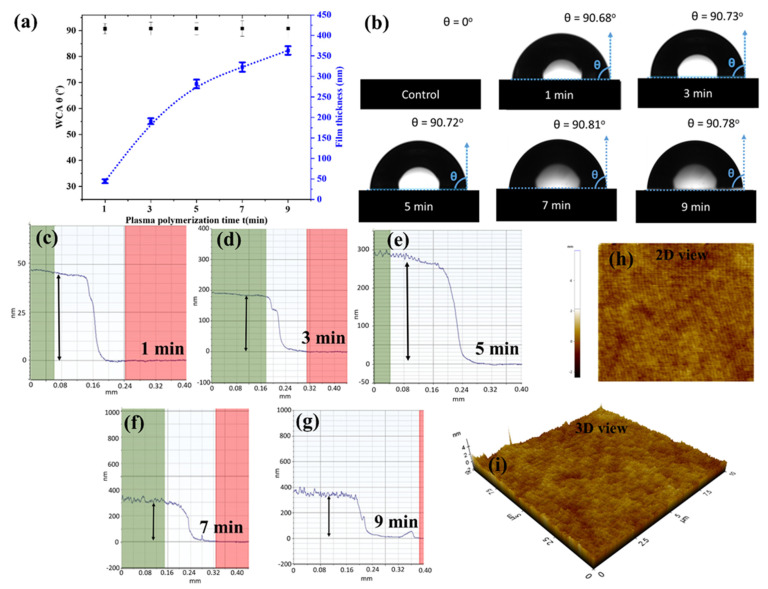
(**a**) Average static WCA and film thickness at different plasma polymerisation times. (**b**) Photographs of WCA of control and AP−PP−lim samples at different plasma polymerisation times. (**c**–**g**) Step height measurements of AP−PP−lim films at different plasma polymerisation times with their corresponding height profile plots. (**h**,**i**) 2D and 3D AFM profiles of the smooth AP−PP−lim nano-thin films deposited at 1 min.

Figure 2a exhibits the average static WCA and thickness of AP-PP-lim nano-thin films deposited on the glass substrate at different plasma polymerisation times. The results were compared with the control sample, a clean glass substrate, which is completely wettable (i.e., WCA = 0°). After the plasma polymerisation at a minimum period of 1 min, a much higher static WCA was obtained at about 90.7 ± 1.1°. No significant changes in WCA were further observed regardless of plasma polymerisation time. The corresponding photographs of water drops on the control and AP-PP-lim samples are shown in Figure 2b. 

From the profilometer measurements, as shown in Figure 2c–g, the thickness of AP-PP-lim deposited at 1, 3, 5, 7, and 9 min was estimated to be about 44.3 ± 4.8, 190.7 ± 7.3, and 281.6 ± 10.5, 322.8 nm ± 11.6 and 363.3 nm ± 10.3 nm, respectively. Meanwhile, no significant deviations of the film thickness were observed across the deposited films, for each deposition time, after plasma polymerisation. Furthermore, the surface morphologies of AP-PP-lim nano-thin films were also characterised based on the topographical AFM measurements (2-D and 3-D view), as shown in Figure 2h,i and Figure S1a–d. The results showed a relationship between the film thickness and plasma polymerisation time with a deposition rate of ~0.8 nm s^−1^, indicating the reproducibility of the plasma polymerisation process when carried out in a controlled manner. By adjusting the plasma polymerisation time with the suggested atmospheric plasma method, one may control the resultant film thickness.

The AFM profiles showed a smooth, and complete coverage of AP-PP-lim (1 min) on the substrate, which correlates well with low average roughness (*R*_a_) value (0.23 ± 0.02 nm) and root mean square roughness (*R_q_*) value (0.27 ± 0.02 nm). The average roughness (*R*_a_) and root mean square roughness (*R_q_*) of AP-PP-lim nano-thin films (3, 5, 7 and 9 min) and control clean glass substrates are shown in Table S1 and Figure S1e. The results showed a linear relationship between the film roughness and plasma polymerisation time. Figures S2 and S3 showed that the nano-thin films deposited at various times on glass substrate are optically transparent. In this work, the *R*_a_ value of AP-PP-lim was comparable to that of low-pressure plasma-polymerised D-limonene reported previously [38].

In comparison, the thickness control in this work was almost comparable to the plasma polymerisation materials reported in the literature, in which the thickness and plasma polymerisation time were likewise linearly related due to a constant flow of precursors [48]. Meanwhile, much thicker films were found to be less effective in antibiofouling activity, possibly due to increased roughness that enhanced the attachment of bacteria on the surfaces [49]. Furthermore, plasma-polymerised coatings with hydrophobic or superhydrophobic surfaces could also greatly reduce the adhesion of bacteria on the surfaces [50]. Hence, in this work, the AP-PP-lim nano-thin films with adequate surface characteristics suitable for antimicrobial activity, deposited at a short period of 1 min, were considered for further characterisations.

**Figure 3:**
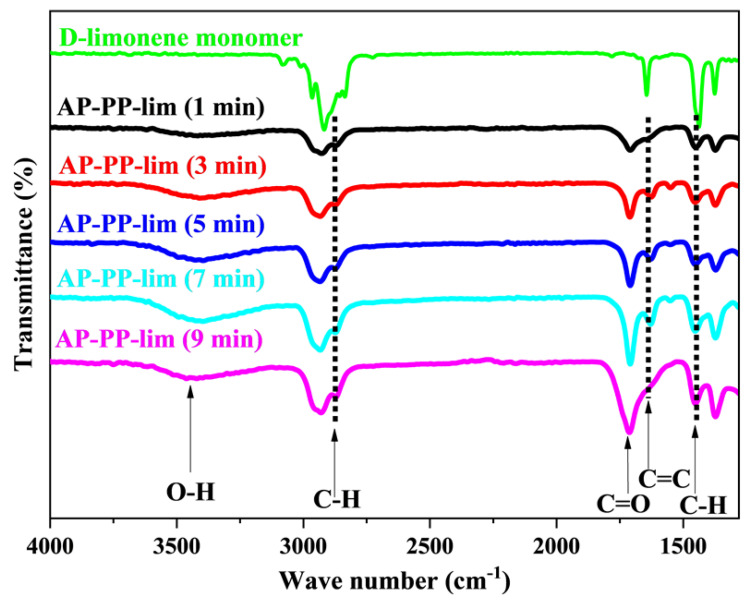
ATR-FTIR spectra of D-limonene monomer and AP−PP−lim deposited on a glass substrate at plasma polymerisation times of 1, 3, 5, 7 and 9 min.

The ATR-FTIR spectra of AP-PP-lim nano-thin films and D-limonene monomer samples are shown in Figure 3 and Figure S4. The molecules of D-limonene consist of C−H, C−C, and C=C bonds [38,51]. Bands include symmetric stretching of C−H bonds (2834 and 2856 cm^−1^), asymmetric stretching of C−H (2920 and 2964 cm^−1^), and unsaturated C−H bonds (3010, 3046, 3072, and 3083 cm^−1^). The stretching band of the C=C bonds was seen at around 1644 cm^−1^. Asymmetric C−H bending was presented at 1436 and 1451 cm^−1^, while symmetric C−H bending was at 1375 cm^−1^. Furthermore, out-of-plane bending of C−H bond was presented at 1310 cm^−1^ as different band with low intensities.

Compared to the monomer D-limonene, the AP-PP-lim nano-thin films, in the surface phase, have broader and fewer noticeable bands because of the cross-linking nature of plasma polymers [52]. Based on Figure 3, AP-PP-lim nano-thin films show the following bands: (1647 cm^−1^) represents unsaturated C=C stretching, (2926 and 2956 cm^−1^) represent asymmetric and symmetric C−H stretching, (1374 and 1450 cm^−1^) represent symmetric and asymmetric bending of C−H. Hence, the cross-linking properties of AP-PP-lim nano-thin films, with random bonding and cross-linking, might be associated with a variety of bonding environments [53,54].

Although the precursor molecule lacks oxygenated chemical groups, polar functionality on plasma-polymerised films arises when they come into contact with ambient air [55]. In atmospheric plasma, the free radicals in the plasma-polymerised films are often produced by plasma active species ablation or imperfect fragment bonding [56]. During the plasma polymerisation, these radicals rapidly interact with oxygen in the air to produce oxygenated species [57]. It is well-known that limonene easily oxidises to carveol, carvone and limonene oxide (Figure S5).

The present authors had already provided plausible mechanisms of plasma polymerisation followed by deposition of carvone on the surface [6]. Similar mechanisms would likely also hold for carveol and limonene oxide to polymerise and deposit on the surface. For instance, a band that arose with a weak intensity at about 1050 cm^−1^, might have originated from the C−O bonds, while a band at about 1709 cm^−1^ was ascribed to the stretching of the C=O bonds that emerge due to similar causes. Another weak broadband at about 3340 cm^−1^ was also presented which corresponds to O−H stretching. Those chemical fingerprints often resemble the existing plasma-polymerised materials produced from essential oils and their constituents [48].

Importantly, the band presented in the AP-PP-lim nano-thin films at about 1647 cm^−1^ corresponds to the C=C bond. The unconjugated C=O bond appeared at 1709 cm^−1^. Both C=O and C=C peaks were similar to the carvone-based system, as reported earlier. Though the C=C peak was often absent in the films prepared with various natural precursors as well as precursors that formally containing double bonds [57,58,59], these authors had reported that for carvone moiety this peak appears almost at the same position for both pure compound and plasma polymerised compound [6]. This result suggested that under the deposition circumstances utilised in this work, chemical structures are partially retained during plasma polymerisation [60]. Furthermore, the same FTIR spectra were also found for the samples deposited at 3, 5, 7, and 9 min, suggesting the same characteristics of chemical bonding regardless of plasma polymerisation time (see Figure 3 and Figure S4). As the outermost surface is always the same for all five deposited surfaces, the WCA is also the same, as shown in Figure 2. Moreover, it might be interesting to note that, as the WCA of the films are ~90°, the variation in the surface roughness does not influence WCA according to Wenzel equation [61].

θm = cos^−1^ (r.cosθY)

where θ_m_ is the measured contact angle, θ_Y_ is the Young contact angle as defined for an ideal surface. r is the roughness ratio. It is defined as the ratio of true area of the solid surface to the apparent area. r = 1 for a smooth surface and >1 for a rough one. As θ_Y_ is ~90°, θ_m_ also become the same ~90° irrespective of r.

**Figure 4:**
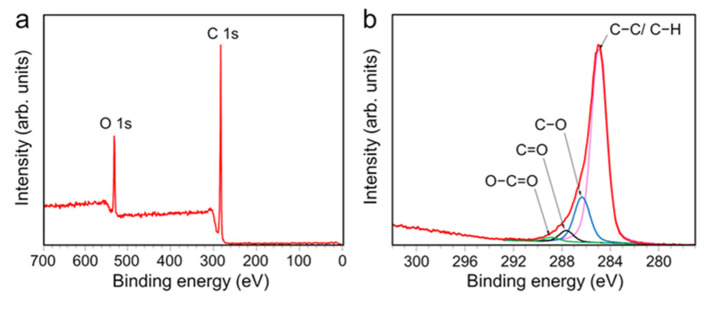
XPS of AP-PP-lim nano-thin films. (**a**) XPS survey spectrum and (**b**) core-level of C 1 s spectra.

XPS analysis was used to evaluate the surface chemical composition of the AP-PP-lim films and confirm the possible chemical bonding on the surfaces. Figure 4a shows an XPS survey spectrum of AP-PP-lim films without any Si signal, thus suggesting that the plasma coating has completely masked the underlying substrate. The results of the XPS survey scan showed typical two C 1 s and O 1 s peaks at around 285 and 533 eV binding energies, respectively. It was observed that the intensity of the C 1 s peak is much higher than that of the O 1 s peak, indicating the highly preserved structure of C=C and C−H bonds after the plasma polymerisation. Considering the atomic concentrations of the AP-PP-lim films, carbon emerged as the dominant content with 84.7 atomic percent, whereas the remaining contribution to the oxygen content is 15.3 atomic percent.

The analysis of the C 1 s peak provided more detailed information about the chemical composition of AP-PP-lim (Figure 4b). The C 1 s peak could be deconvoluted into four components: hydrocarbons bonds (C−C/C−H) with a binding energy of 284.8 eV form the polymer backbone; and chemical groups such as hydroxyl (C−O–H)/ether (C–O–C), carbonyl (C=O), and carboxyl (O−C=O) with binding energies of 286.4 eV, 287.7 eV, and 289.0 eV, respectively, are incorporated in the polymer matrix [58]. The existence of C−O, C=O, and O−C=O bonds indicates that AP-PP-lim has been partially oxidised, and the findings correlated well with the FTIR spectra.

These findings were consistent with previous research on plasma-polymerised natural oils [58,62]. The relative concentrations of distinct carbon bonding states in relation to the overall carbon concentration in the sample was estimated using Gaussian curve fitting. Hydrocarbon species (79.1% of total carbon) formed the backbone of the coatings and reflected the chemical bonds of D-limonene. Compared to prior studies employing AP plasma [63], the quantity of hydrocarbons (C−C/C−H) dominated this coating composition. The second largest contribution came from C−O groups, which accounted for 16.5% of the total, with the remaining 3.0% and 1.4% contributed by C=O and O−C=O groups, respectively.

**Figure 5:**
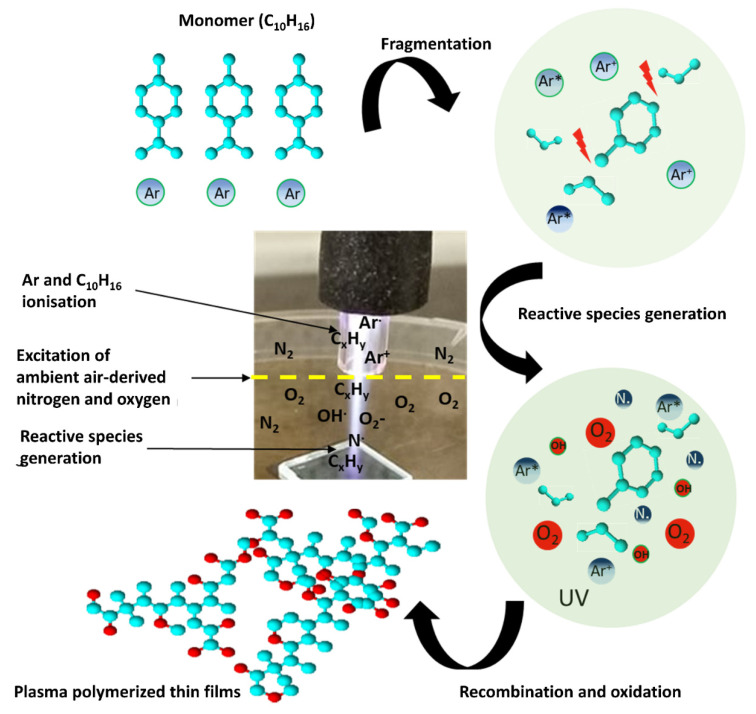
Schematic representation of plasma polymer derived from D-limonene monomer using the APPJ system.

A proposed mechanism of AP-PP-lim chemical structure based on the ATR-FTIR and XPS analysis is shown in Figure 5. The plasma produced reactive species by ionising a flow of argon gas and D-limonene (C_10_H_16_) vapour that subsequently reacted with ambient air to form gas-phase reactive hydrocarbon (C*_x_*H*_y_*), oxygen (O), and nitrogen (N) species, respectively. During the dissociation in plasma, the C_10_H_16_ monomers partially broke into fragments that consisted of ions, radicals, and molecules. Due to its unique reaction process with ambient air, the fragments reacted with the oxygen reactive species and recombined onto the substrate to create a highly cross-linked polymer with new oxygen chemical groups (e.g., C−O, C=O, and O−C=O). In this work, the new chemical groups indicated the typical characteristics of plasma-polymerised nano-thin films that behaved similarly to the previous work conducted at low-pressure [38].

### 3.2. Antimicrobial Performance of AP-PP-lim Nano-Thin Films

Antibacterial properties of D-limonene have attracted great interest in bio-fouling studies since it is a bio-safe essential oil that can act as an excellent antimicrobial agent [64]. It was also well known that D-limonene has superb antimicrobial properties to different species of bacteria strains such as *E. coli* and *Staphylococcus aureus* (*S. aureus*). Recent studies by Gupta et al. and Han et al. also reported that D-limonene possesses excellent antibacterial properties to prevent different kinds of bacterial propagation and their growth [7,65]. In this work, Gram-negative (*E. coli*) bacteria (which cause biofouling) were used to examine the antibacterial performance of the AP-PP-lim nano-thin films. Gram-negative bacteria, unlike Gram-positive bacteria, tend to be antibiotic resistance due to their thick cell walls [66]. As a result, if a coating is resistant to Gram-negative bacteria, it is likely to be resistant to Gram-positive bacterial growth as well.

**Figure 6:**
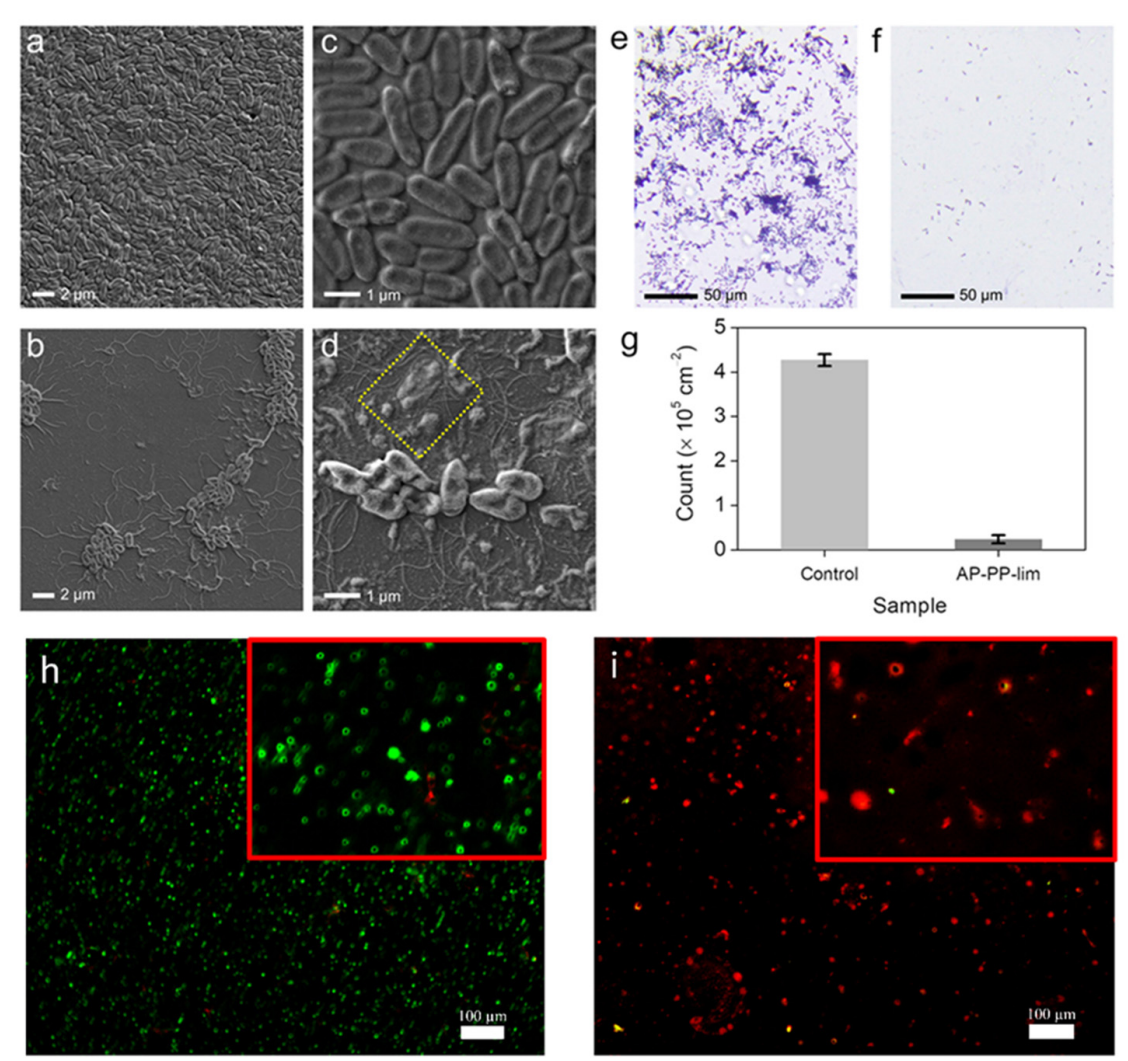
Antimicrobial performance of AP−PP−lim nano-thin films against Gram-negative (*E. coli*) bacteria. FESEM images of treated *E. coli* on the (**a**,**c**) control and (**b**,**d**) AP−PP−lim sample. The yellow square box indicates the rupture of *E. coli* cells. Fluorescent microscopic imaging of treated crystal violet-stained E. coli on the (**e**) control and (**f**) AP−PP−lim sample. (**g**) Count of treated *E. coli* per unit area (cm^−2^) on the control and AP−PP−lim sample. Illustrative fluorescence views in live-dead fluorescence assay of (**h**) *E. coli* attached on clean glass substrate and (**i**) *E. coli* attached on AP−PP−lim. Samples were incubated for 24 h. Live bacteria are represented by green, whereas dead bacteria are represented by red.

The mechanisms by which contact-active antibacterial coatings affect cell death are often associated with disruption of the cell surface [67,68,69]. To examine this hypothesis, the morphologies of *E. coli* coated onto the control and treated samples with AP-PP-lim nano-thin films were imaged by FESEM. Prior to the evaluation, both control and AP-PP-lim samples were separately incubated with 10^5^ CFU mL^−1^ of *E. coli* cell suspension for 5 h, then the cells underwent fixation, and substrates were treated with glutaraldehyde (2.5%), followed by ethanol (20–100%) dehydration and air drying. Figure 6a−d shows the FESEM images of the morphological changes in the treated *E. coli* between the control and AP-PP-lim sample. It was observed that the typical rod-shaped *E. coli* bacteria (about 2.00 μm long and 0.25−1.00 μm wide) on the control sample incubated with the cell suspension have remained almost unchanged. The *E. coli* on the control sample grew well with intact cytoplasmic membranes and formed abundant biofilms (Figure 6a,c).

In contrast, the treated *E. coli* on the AP-PP-lim were found to be isolated with a significant decrease in the bacterial colonies, as shown in Figure 6b. The shape of *E. coli* was shrivelled, which indicates that the AP-PP-lim could distort the bacteria upon their initial attachment on the surface. Interestingly, further rupture to the structure of dead *E. coli* was apparently seen in the yellow box of Figure 6d, indicating that the AP-PP-lim could disrupt and collapse the cytoplasmic membranes through the affected pores, leading to death. It was noted that the bacterial outer cover (cell membrane or cell wall) is the most probable cellular goal for D-limonene due to the creation of pores. Furthermore, lipids in the cell wall composition from numerous bacterial classes explained their various vulnerabilities to D-limonene. In this case, the findings suggested that the AP-PP-lim nano-thin films not only inhibit the propagation and growth of bacteria, but also possess a direct bactericidal effect.

To analyse the bacteria count, the fluorescence effects of biofilm assay and early attachment assay were quantitatively evaluated. Figure 6e,f shows the fluorescence microscopy images of treated *E. coli* bacteria adhered onto the control and AP-PP-lim sample after 24 h of incubation. It was revealed that the number of bacteria adhered onto the AP-PP-lim sample was much lower (with a count of 2.37 × 10^4^ ± 0.9 × 10^3^ cm^−2^) than that of the control sample (with a count of 4.27 × 10^5^ ± 1.3 × 10^4^ cm^−2^) (see Figure 6g). The findings suggest that the AP-PP-lim nano-thin films significantly contribute to the reduction in bacterial adhesion by up to 94% as compared to the control sample. The initial adhesion assay and the biofilm assay findings for fluorescence were qualitatively evaluated. Propidium iodide stains dead bacteria red by penetrating their ruptured membrane, while SYTO9 turned live bacteria green. After being incubated for 24 h, the fluorescence pictures of *E. coli*’s initial attachment to a clean glass substrate and an AP-PP-lim are shown in Figure 6h and Figure 6i, respectively. AP-PP-lim demonstrated a much lower amount of *E. coli* adhered in comparison to a clean glass substrate and dead bacteria in red colour are visible, as shown in Figure 6i. It should be noted that the biofilm adhesion could be influenced by more factors than just surface reactivity. Variations in biofilm surface adherence may be due to the synergetic effects of multiple factors (e.g., reduced adhesion or destruction of intercellular signal conduction and communication), which might affect the contact of bacteria onto the surface, according to the mechanism reported previously [70].

## 4. Conclusions

In summary, optically transparent and smooth AP-PP-lim nano-thin films as an antibacterial coating were successfully deposited on glass substrates using the precursor D-limonene via a rapid and single-step APPP method. The physicochemical properties of the AP-PP-lim nano-thin films were systematically investigated in relation to the plasma polymerisation time. Under the circumstances utilised in this work, a high deposition rate of ~0.8 nm s^−1^ can be achieved to control the thickness of AP-PP-lim nano-thin films. Surface chemical analysis of the resultant nano-thin films indicated that the hydrocarbon molecules (e.g., C−C/ C−H) are partly retained from the fragmented D-limonene, along with new oxygen chemical groups (e.g., C−O, C=O, and O−C=O), resulting from the free radical reactions in ambient air under the plasma condition. For its antimicrobial behaviour, the biofilm assay evaluation exhibited a 94% decrease in the number of *E. coli,* compared to the control sample, suggesting the effectiveness of the AP-PP-lim nano-thin films as an antibacterial coating against Gram-negative bacteria. Overall, this APPP method can provide a new insight into the development of bacteria-resistant and biocompatible coatings based on precursors.

## Data Availability

The data used to support the findings of this study are available from the corresponding author upon request.

## Supplementary Materials

The following supporting information can be downloaded at: https://www.mdpi.com/article/10.3390/polym15020307/s1. Figure S1: 2D and 3D AFM profiles of the smooth AP-PP-lim nano-thin films deposited were at 3-, 5-, 7- and 9-min; Figure S2: Transmittance and absorption spectra of AP-PP-lim thin films with different deposition time (1-, 3-, and 5-min) deposited on a glass substrate; Figure S3: Photograph of a glass substrate with optically transparent AP-PP-lim nano-thin films; Figure S4: ATR-FTIR spectra of pure D-limonene and the AP-PP-lim films at 1-, 3-, 5-, 7- and 9-min plasma polymerisation; Figure S5: Chemical structure of D-limonene and its oxidation products; Table S1: Average roughness (R_a_) and root mean square roughness (R_q_) of glass substrate and AP-PP-lim nano thin films were deposited at 3-, 5-. 7- and 9-min.

## References

[1] Barros C.H., Casey E. (2020). A Review of Nanomaterials and Technologies for Enhancing the Antibiofilm Activity of Natural Products and Phytochemicals. ACS Appl. Nano Mater..

[2] Kosová K., Vítámvás P., Urban M.O., Prášil I.T., Renaut J. (2018). Plant Abiotic Stress Proteomics: The Major Factors Determining Alterations in Cellular Proteome. Front. Plant Sci..

[3] Rodríguez-Casanovas H.J., la Rosa M.D., Bello-Lemus Y., Rasperini G., Acosta-Hoyos A.J. (2022). Virucidal Activity of Different Mouthwashes Using a Novel Biochemical Assay. Healthcare.

[4] Prabuseenivasan S., Jayakumar M., Ignacimuthu S. (2006). In Vitro Antibacterial Activity of Some Plant Essential Oils. BMC Complement. Altern. Med..

[5] Burt S. (2004). Essential Oils: Their Antibacterial Properties and Potential Applications in Foods—A Review. Int. J. Food Microbiol..

[6] Masood A., Ahmed N., Mohd Razip Wee M.F., Haniff M.A.S.M., Mahmoudi E., Patra A., Siow K.S. (2022). Pulsed Plasma Polymerisation of Carvone: Characterisations and Antibacterial Properties. Surf. Innov..

[7] Gupta A., Jeyakumar E., Lawrence R. (2021). Strategic Approach of Multifaceted Antibacterial Mechanism of Limonene Traced in Escherichia Coli. Sci. Rep..

[8] Hetrick E.M., Schoenfisch M.H. (2006). Reducing Implant-Related Infections: Active Release Strategies. Chem. Soc. Rev..

[9] Hickok N.J., Shapiro I.M. (2012). Immobilized Antibiotics to Prevent Orthopaedic Implant Infections. Adv. Drug Del. Rev..

[10] Inzana J.A., Schwarz E.M., Kates S.L., Awad H.A. (2016). Biomaterials Approaches to Treating Implant-Associated Osteomyelitis. Biomaterials.

[11] Cong Y., Quan C., Liu M., Liu J., Huang G., Tong G., Yin Y., Zhang C., Jiang Q. (2015). Alendronate-Decorated Biodegradable Polymeric Micelles for Potential Bone-Targeted Delivery of Vancomycin. J. Biomater. Sci. Polym. Ed..

[12] Wang Y., Li P., Xiang P., Lu J., Yuan J., Shen J. (2016). Electrospun Polyurethane/Keratin/Agnp Biocomposite Mats for Biocompatible and Antibacterial Wound Dressings. J. Mater. Chem. B.

[13] Fang B., Jiang Y., Nüsslein K., Rotello V.M., Santore M.M. (2015). Antimicrobial Surfaces Containing Cationic Nanoparticles: How Immobilized, Clustered, and Protruding Cationic Charge Presentation Affects Killing Activity and Kinetics. Colloids Surf. B. Biointerfaces.

[14] Fuchs A.D., Tiller J.C. (2006). Contact-Active Antimicrobial Coatings Derived from Aqueous Suspensions. Angew. Chem. Int. Ed..

[15] Milović N.M., Wang J., Lewis K., Klibanov A.M. (2005). Immobilized N-Alkylated Polyethylenimine Avidly Kills Bacteria by Rupturing Cell Membranes with No Resistance Developed. Biotechnol. Bioeng..

[16] Förch R., Chifen A.N., Bousquet A., Khor H.L., Jungblut M., Chu L.Q., Zhang Z., Osey-Mensah I., Sinner E.K., Knoll W. (2007). Recent and Expected Roles of Plasma-Polymerized Films for Biomedical Applications. Chem. Vap. Deposition.

[17] Franklin A.D. (2015). Nanomaterials in Transistors: From High-Performance to Thin-Film Applications. Science.

[18] Montemor M.d.F. (2014). Functional and Smart Coatings for Corrosion Protection: A Review of Recent Advances. Surf. Coat. Technol..

[19] Zare M., Zare M., Butler J.A., Ramakrishna S. (2021). Nanoscience-Led Antimicrobial Surface Engineering to Prevent Infections. ACS Appl. Nano Mater..

[20] Lishchynskyi O., Shymborska Y., Stetsyshyn Y., Raczkowska J., Skirtach A.G., Peretiatko T., Budkowski A. (2022). Passive Antifouling and Active Self-Disinfecting Antiviral Surfaces. Chem. Eng. J..

[21] Schröder K., Finke B., Ohl A., Lüthen F., Bergemann C., Nebe B., Rychly J., Walschus U., Schlosser M., Liefeith K. (2010). Capability of Differently Charged Plasma Polymer Coatings for Control of Tissue Interactions with Titanium Surfaces. J. Adhes. Sci. Technol..

[22] Cao N., Miao Y., Zhang D., Boukherroub R., Lin X., Ju H., Li H. (2018). Preparation of Mussel-Inspired Perfluorinated Polydopamine Film on Brass Substrates: Superhydrophobic and Anti-Corrosion Application. Prog. Org. Coat..

[23] Zhou C., Shi Y., Sun C., Yu S., Liu M., Gao C. (2014). Thin-Film Composite Membranes Formed by Interfacial Polymerization with Natural Material Sericin and Trimesoyl Chloride for Nanofiltration. J. Membr. Sci..

[24] Gupta P., Nayak K.K. (2015). Characteristics of Protein-Based Biopolymer and Its Application. Polym. Eng. Sci..

[25] Woloszyn J.D., Hesse P., Hungenberg K.D., McAuley K.B. (2013). Parameter Selection and Estimation Techniques in a Styrene Polymerization Model. Macromol. React. Eng..

[26] Yasuda H., Bumgarner M., Marsh H., Morosoff N. (1976). Plasma Polymerization of Some Organic Compounds and Properties of the Polymers. J. Polym. Sci., Polym. Chem. Ed..

[27] Kumar A., Al-Jumaili A., Prasad K., Bazaka K., Mulvey P., Warner J., Jacob M.V. (2020). Pulse Plasma Deposition of Terpinen-4-Ol: An Insight into Polymerization Mechanism and Enhanced Antibacterial Response of Developed Thin Films. Plasma Chem. Plasma Process..

[28] Al-Jumaili A., Bazaka K., Jacob M.V. (2017). Retention of Antibacterial Activity in Geranium Plasma Polymer Thin Films. Nanomaterials.

[29] Chan Y.W., Siow K.S., Ng P.Y., Gires U., Majlis B.Y. (2016). Plasma Polymerized Carvone as an Antibacterial and Biocompatible Coating. Mater. Sci. Eng. C.

[30] Sharifahmadian O., Zhai C., Hung J., Shineh G., Stewart C.A.C., Fadzil A.A., Ionescu M., Gan Y., Wise S.G., Akhavan B. (2021). Mechanically Robust Nitrogen-Rich Plasma Polymers: Biofunctional Interfaces for Surface Engineering of Biomedical Implants. Mater. Today Adv..

[31] Bhatt S., Pulpytel J., Arefi-Khonsari F. (2015). Low and Atmospheric Plasma Polymerisation of Nanocoatings for Bio-Applications. Surf. Innov..

[32] Deng X., Leys C., Vujosevic D., Vuksanovic V., Cvelbar U., De Geyter N., Morent R., Nikiforov A. (2014). Engineering of Composite Organosilicon Thin Films with Embedded Silver Nanoparticles Via Atmospheric Pressure Plasma Process for Antibacterial Activity. Plasma Process. Polym..

[33] Khan M., Rehman N., Khan S., Ullah N., Masood A., Ullah A. (2019). Spectroscopic Study of Co2 and Co2–N2 Mixture Plasma Using Dielectric Barrier Discharge. AIP Adv..

[34] Lou B.-S., Lai C.-H., Chu T.-P., Hsieh J.-H., Chen C.-M., Su Y.-M., Hou C.-W., Chou P.-Y., Lee J.-W. (2019). Parameters Affecting the Antimicrobial Properties of Cold Atmospheric Plasma Jet. J. Clin. Med. Res..

[35] Ma C., Nikiforov A., De Geyter N., Morent R., Ostrikov K.K. (2022). Plasma for Biomedical Decontamination: From Plasma-Engineered to Plasma-Active Antimicrobial Surfaces. Curr. Opin. Chem. Eng..

[36] Izadjoo M., Zack S., Kim H., Skiba J. (2018). Medical Applications of Cold Atmospheric Plasma: State of the Science. J. Wound Care.

[37] Jungbauer G., Moser D., Müller S., Pfister W., Sculean A., Eick S. (2021). The Antimicrobial Effect of Cold Atmospheric Plasma against Dental Pathogens—A Systematic Review of in-Vitro Studies. Antibiotics.

[38] Gerchman D., Bones B., Pereira M., Takimi A. (2019). Thin Film Deposition by Plasma Polymerization Using D-Limonene as a Renewable Precursor. Prog. Org. Coat..

[39] Siow K.S., Britcher L., Kumar S., Griesser H. (2019). Qcm-D and Xps Study of Protein Adsorption on Plasma Polymers with Sulfonate and Phosphonate Surface Groups. Colloids Surf. B. Biointerfaces.

[40] Ahmed N., Masood A., Siow K.S., Wee M., Haron F.F., Patra A., Nayan N., Soon C.F.J.P.C., Processing P. (2022). Effects of Oxygen (O2) Plasma Treatment in Promoting the Germination and Growth of Chili. Plasma Chem. Plasma Process..

[41] Madkour A.E., Tew G.N. (2008). Towards Self-Sterilizing Medical Devices: Controlling Infection. Polym. Int..

[42] Moyes R.B., Reynolds J., Breakwell D.P. (2009). Differential Staining of Bacteria: Gram Stain. Curr. Protoc. Microbiol..

[43] Wickramasinghe S., Ju M., Milbrandt N.B., Tsai Y.H., Navarreto-Lugo M., Visperas A., Klika A., Barsoum W., Higuera-Rueda C.A., Samia A.C.S. (2020). Photoactivated Gold Nanorod Hydrogel Composite Containing D-Amino Acids for the Complete Eradication of Bacterial Biofilms on Metal Alloy Implant Materials. ACS Appl. Nano Mater..

[44] Srinivasan S., McKinley G.H., Cohen R.E. (2011). Assessing the Accuracy of Contact Angle Measurements for Sessile Drops on Liquid-Repellent Surfaces. Langmuir.

[45] Wang Y., Sang D.K., Du Z., Zhang C., Tian M., Mi J. (2014). Interfacial Structures, Surface Tensions, and Contact Angles of Diiodomethane on Fluorinated Polymers. J. Phys. Chem. C.

[46] Belibel R., Avramoglou T., Garcia A., Barbaud C., Mora L. (2016). Effect of Chemical Heterogeneity of Biodegradable Polymers on Surface Energy: A Static Contact Angle Analysis of Polyester Model Films. Mater. Sci. Eng. C.

[47] Fahmy A., Mix R., Schönhals A., Friedrich J. (2012). Surface and Bulk Structure of Thin Spin Coated and Plasma-Polymerized Polystyrene Films. Plasma Chem. Plasma Process..

[48] Jacob M.V., Easton C.D., Anderson L.J., Bazaka K. (2014). Rf Plasma Polymerised Thin Films from Natural Resources. Int. J. Mod. Phys. Conf. Ser..

[49] Zhao C., Li L., Wang Q., Yu Q., Zheng J. (2011). Effect of Film Thickness on the Antifouling Performance of Poly (Hydroxy-Functional Methacrylates) Grafted Surfaces. Langmuir.

[50] Yuan Y., Hays M.P., Hardwidge P.R., Kim J. (2017). Surface Characteristics Influencing Bacterial Adhesion to Polymeric Substrates. RSC Adv..

[51] Derdar H., Belbachir M., Harrane A. (2019). A Green Synthesis of Polylimonene Using Maghnite-H+, an Exchanged Montmorillonite Clay, as Eco-Catalyst. Bull. Chem. React. Eng. Catal..

[52] Siow K.S., Britcher L., Kumar S., Griesser H.J. (2017). Plasma Polymers Containing Sulfur and Their Co-Polymers with 1, 7-Octadiene: Chemical and Structural Analysis. Plasma Process. Polym..

[53] Palaniappan S., Narayana B. (1994). Temperature Effect on Conducting Polyaniline Salts: Thermal and Spectral Studies. J. Polym. Sci. Part A Polym. Chem..

[54] Burkey D.D., Gleason K.K. (2003). Structure and Thermal Properties of Thin Film Poly (A-Methylstyrene) Deposited Via Plasma-Enhanced Chemical Vapor Deposition. Chem. Vap. Depos..

[55] Easton C., Jacob M. (2009). Ageing and Thermal Degradation of Plasma Polymerised Thin Films Derived from Lavandula Angustifolia Essential Oil. Polym. Degrad. Stab..

[56] Clouet F., Shi M. (1992). Interactions of Polymer Model Surfaces with Cold Plasmas: Hexatriacontane as a Model Molecule of High-Density Polyethylene and Octadecyl Octadecanoate as a Model of Polyester. I. Degradation Rate Versus Time and Power. J. Appl. Polym. Sci..

[57] Bazaka K., Jacob M.V. (2010). Post-Deposition Ageing Reactions of Plasma Derived Polyterpenol Thin Films. Polym. Degrad. Stab..

[58] Alancherry S., Bazaka K., Jacob M.V. (2018). Rf Plasma Polymerization of Orange Oil and Characterization of the Polymer Thin Films. J. Polym. Environ..

[59] Bazaka K., Jacob M.V., Shanks R.A. (2010). Fabrication and Characterization of Rf Plasma Polymerized Thin Films from 3, 7-Dimethyl-1, 6-Octadien-3-Ol for Electronic and Biomaterial Applications. Adv. Mater. Res..

[60] Friedrich J. (2011). Mechanisms of Plasma Polymerization–Reviewed from a Chemical Point of View. Plasma Process. Polym..

[61] Drábik M., Polonskyi O., Kylián O., Čechvala J., Artemenko A., Gordeev I., Choukourov A., Slavínská D., Matolínová I., Biederman H. (2010). Super-Hydrophobic Coatings Prepared by Rf Magnetron Sputtering of Ptfe. Plasma Process. Polym..

[62] Ahmad J., Bazaka K., Whittle J.D., Michelmore A., Jacob M.V. (2015). Structural Characterization of Γ-Terpinene Thin Films Using Mass Spectroscopy and X-Ray Photoelectron Spectroscopy. Plasma Process. Polym..

[63] Park C.-S., Jung E.Y., Kim D.H., Kim D.Y., Lee H.-K., Shin B.J., Lee D.H., Tae H.-S. (2017). Atmospheric Pressure Plasma Polymerization Synthesis and Characterization of Polyaniline Films Doped with and without Iodine. Materials.

[64] Espina L., Gelaw T.K., de Lamo-Castellvi S., Pagán R., Garcia-Gonzalo D. (2013). Mechanism of Bacterial Inactivation by (+)-Limonene and Its Potential Use in Food Preservation Combined Processes. PLoS ONE.

[65] Han Y., Chen W., Sun Z. (2021). Antimicrobial Activity and Mechanism of Limonene against Staphylococcus Aureus. J. Food Saf..

[66] Nikaido H. (1996). Multidrug Efflux Pumps of Gram-Negative Bacteria. J. Bacteriol..

[67] Diu T., Faruqui N., Sjöström T., Lamarre B., Jenkinson H.F., Su B., Ryadnov M.G. (2014). Cicada-Inspired Cell-Instructive Nanopatterned Arrays. Sci. Rep..

[68] Schiffman J.D., Elimelech M. (2011). Antibacterial Activity of Electrospun Polymer Mats with Incorporated Narrow Diameter Single-Walled Carbon Nanotubes. ACS Appl. Mater. Interfaces..

[69] Lee S.B., Koepsel R.R., Morley S.W., Matyjaszewski K., Sun Y., Russell A.J. (2004). Permanent, Nonleaching Antibacterial Surfaces. 1. Synthesis by Atom Transfer Radical Polymerization. Biomacromolecules.

[70] Kumar V., Pulpytel J., Giudetti G., Rauscher H., Rossi F., Arefi-Khonsari F. (2011). Amphiphilic Copolymer Coatings Via Plasma Polymerisation Process: Switching and Anti-Biofouling Characteristics. Plasma Process. Polym..

