# Supplementary material for: Atmospheric Pressure Plasma Polymerisation of D-Limonene and Its Antimicrobial Activity
Source: Polymers (Basel). 2023 Jan 6;15(2):307. doi: 10.3390/polym15020307 (PMC9861354; doi:10.3390/polym15020307)
Supplement: Supplementary file 1 [file polymers-15-00307-s001.zip › polymers-1979802-supplementary.pdf]

Supporting Information: Atmospheric Pressure Plasma Polymerisation of D-Limonene and its Antimicrobial Activity

Asad Masood,[1] Naeem Ahmed,[1] M. F. Mohd Razip Wee,[1] Anuttam Patra,[2] Ebrahim Mahmoudi,[3] and Kim S. Siow [1,*]

1.2. D and 3D AFM profiles of the smooth AP-PP-lim nanothin films deposited were at 3-, 5-, 7- and 9- min.

The surface morphologies of AP-PP-lim (3-, 5-, 7- and 9- min) nanothin films were characterised based on the topographical AFM measurements (2-D and 3-D view), as shown in **Figure S1 (a-d)**. The average roughness and root mean square roughness of bare glass substrate values of 1.32 ± 0.11 and 1.57 ± 0.12 nm respectively. The AFM profiles revealed that the glass substrate was completely covered in AP-PP-lim, with average roughness (R_a) values of 0.27 ± 0.01 nm, 0.41 ± 0.02 nm, 0.49 ± 0.03 nm, and 0.89 ± 0.04nm for 3-, 5-, 7- and 9-min of deposition. The root mean square roughness (R_q) values are measured to be 0.27 ± 0.02 nm, 0.54 ± 0.03 nm, 0.63± 0.03 nm and 0.98 ± 0.05 nm for AP-PP-lim (3, 5, 7, and 9) min respectively. The values show that roughness increased with an increase in deposition time (**Figure S1 (e)**).

Figure S1. (a-d) 2D and 3D AFM images of the AP-PP-lim nanothin films deposited at 3, 5, 7, and 9 min respectively, **(e)** average roughness and root mean square roughness at different plasma polymerisation times.

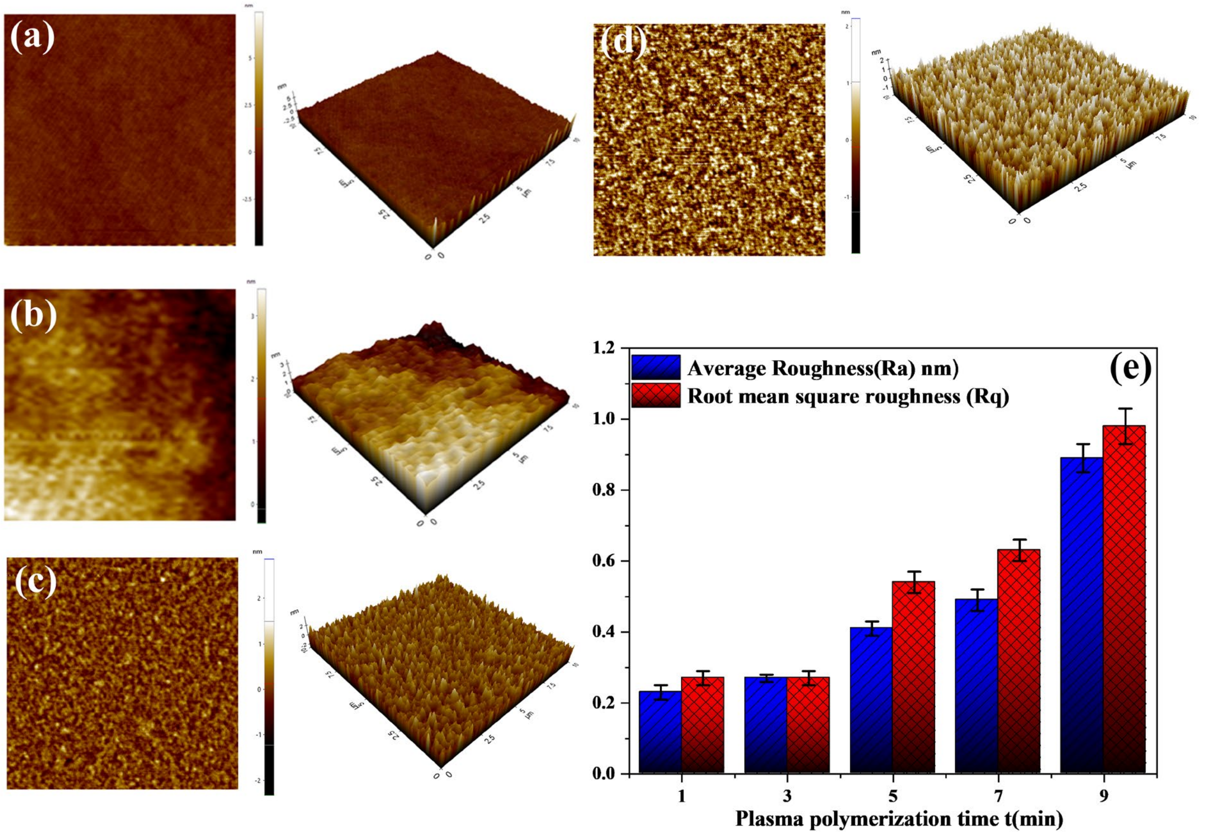

Table S1. Average roughness (R_a) and root mean square roughness (R_q) of glass substrate and AP-PP-lim nano thin films were deposited at 3, 5, 7 and 9 min.

	**Average roughness (R_a) nm**	**Root mean square roughness (R_q) nm**
AP-PP-lim (1 min)	0.23 ± 0.02	0.27 ± 0.02
AP-PP-lim (3 min)	0.27 ± 0.01	0.28 ± 0.02
AP-PP-lim (5 min)	0.41 ± 0.02	0.54 ± 0.03
AP-PP-lim (7 min)	0.49 ± 0.03	0.63 ± 0.03
AP-PP-lim (9 min)	0.89 ± 0.04	0.98 ± 0.05
Glass substrate	1.32 ± 0.11	1.57 ± 0.12

2. UV-Vis Analysis of AP-PP-lim

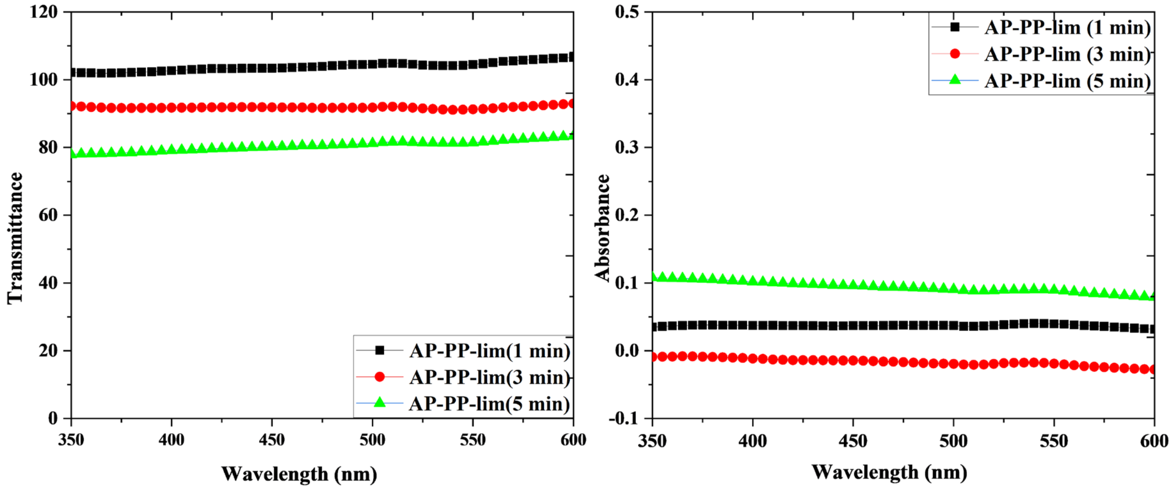

Figure S2. Transmittance spectra and absorption spectra of AP-PP-lim thin films with different deposition time (1 ,3, and 5 min) deposited on a glass substrate.

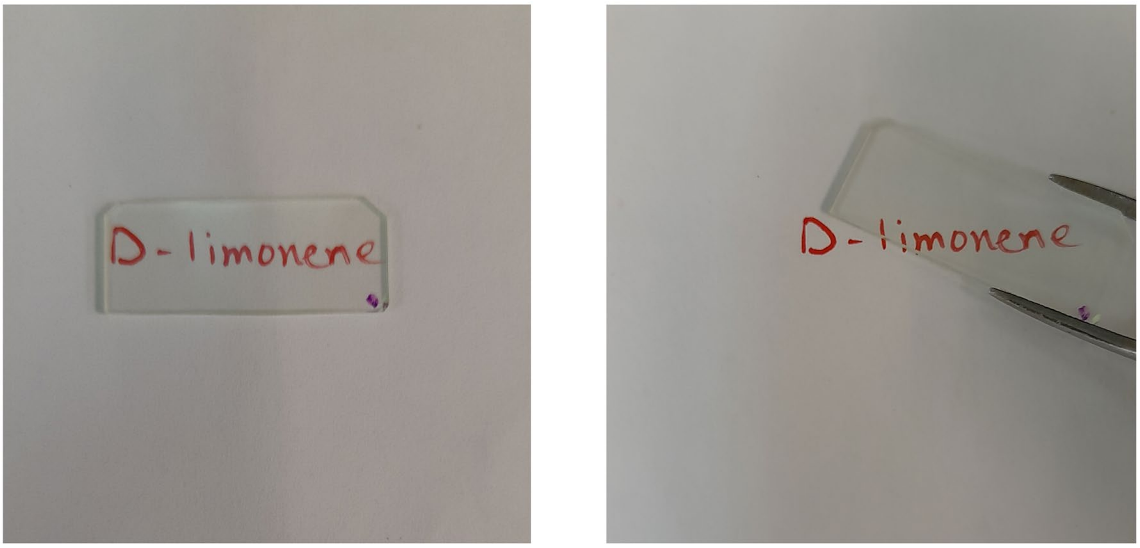

Figure S3. Photograph of a glass substrate with optically transparent AP-PP-lim nano-thin films (1 min).

3. ATR-FTIR of the AP-PP-lim films at 1-, 3-, 5-, 7-, and 9- min plasma polymerisation.

The plasma polymerisation of D-limonene was carried out for different time intervals. Figure S4 showed the ATR-FTIR spectra of AP-PP-lim films deposited for 1-, 3-, 5-, 7-, and 9 min. All spectra results exhibited distinctive bands mostly similar to that of monomer D-limonene (which mainly consists of typical C–H, C–C, and C=C bonds only) (see Figure 3). Few new bands were seen at around 1709 and 3340 cm^{-1}, which correspond to the C=O stretching, and O–H stretching, respectively, resulting from the oxidation in the air during the plasma polymerisation. As no additional peaks appeared for the films deposited for 3-, 5-, 7-, and 9 minutes, as compared to those of AP-PP-lim deposited at 1 min, it can be concluded that chemical structures are the same for all the three films. Therefore, it can be concluded that all (3- ,5-, 7-, and 9-min) plasma polymerisation at atmospheric pressure only differed in their FTIR band intensities, which increased monotonously with an increase of the deposition time.

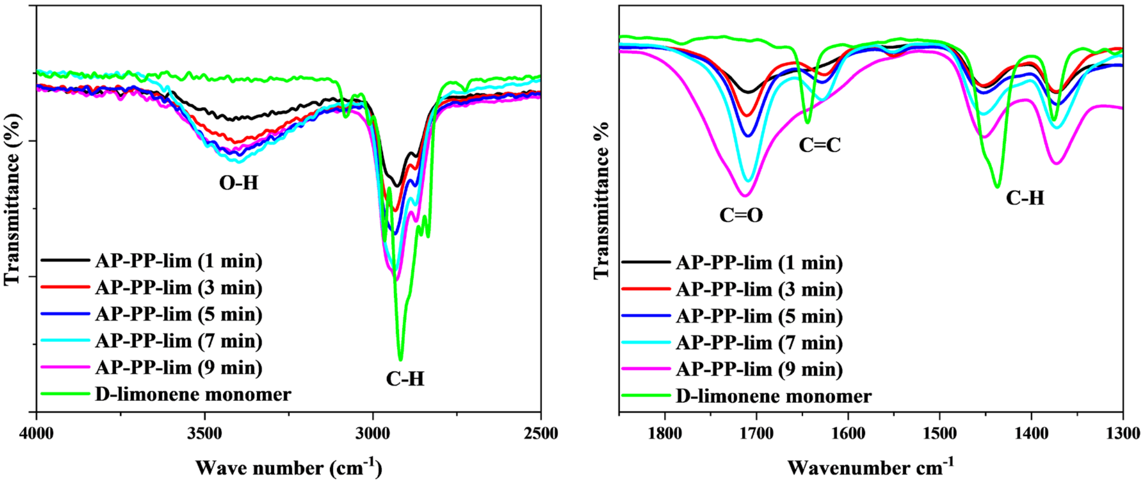

Figure S4. ATR-FTIR spectra of the monomer D-limonene and AP-PP-lim films for 1, 3, 5,7 and 9 min of plasma polymerisation.

4. Chemical structure of D-limonene and its oxidation products.

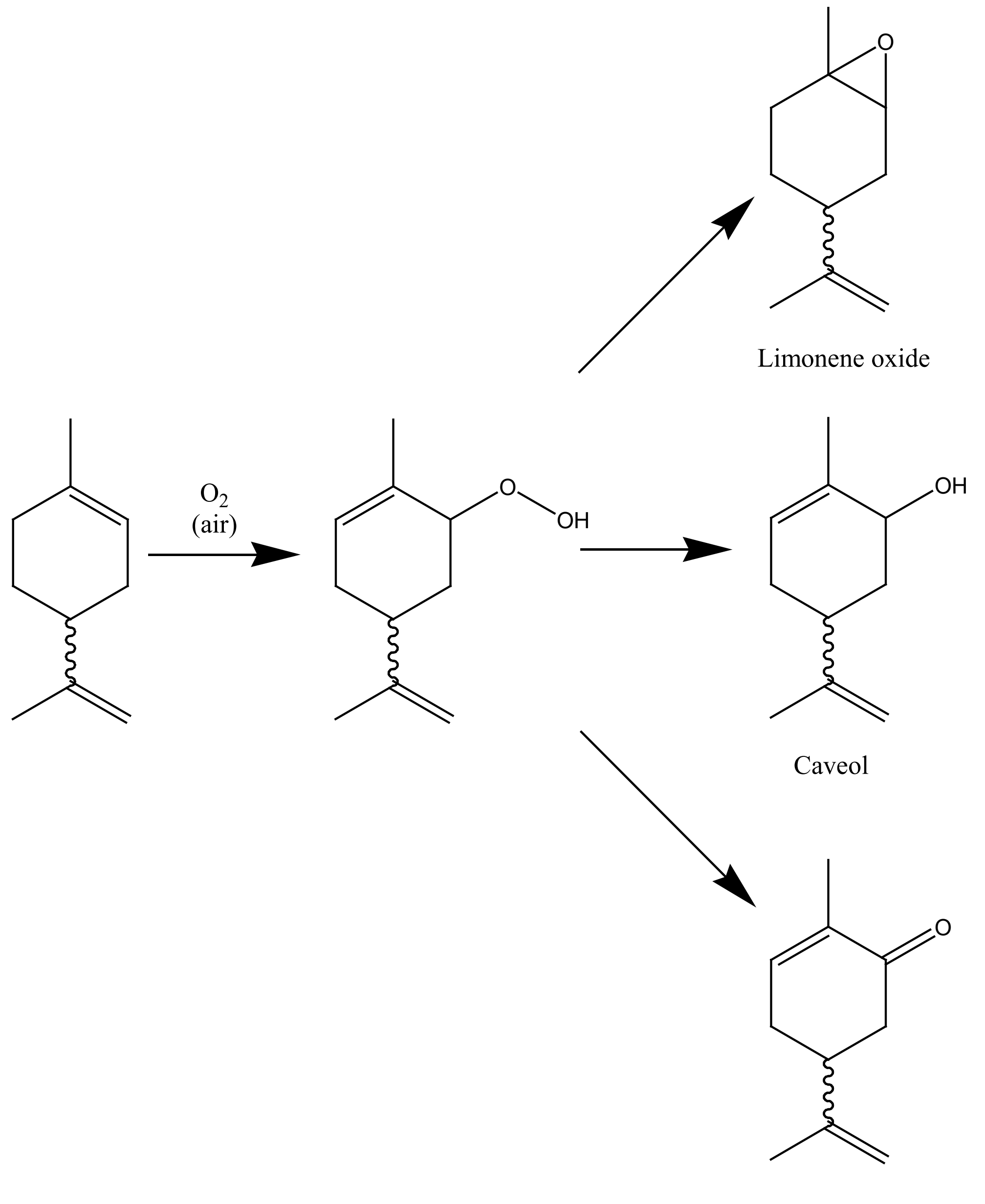

Figure S5. Structure of limonene and its oxidation products.

Limonene can easily be oxidised to limonene oxide, carveol and carvone. The intermediate limonene-2-hydroperoxide is unstable, and quickly transformed to more stable oxidation products mentioned above. Both in FTIRS and XPS, fingerprints of presence of the oxidation products can be detected.
